# Influence of the perceptions of amenities on consumer emotions in urban consumption spaces

**DOI:** 10.1371/journal.pone.0304203

**Published:** 2024-05-29

**Authors:** Jiajie Tan, Yingying Li

**Affiliations:** 1 Shanghai Institute of Tourism, Shanghai Normal University, Shanghai, China; 2 School of Media and Communication, Shanghai Jiaotong University, Shanghai, China; Uniwersytet Gdanski, POLAND

## Abstract

The purpose of this study is to understand how consumers perceive amenities and their impact on promoting positive consumer emotions and comprehend the role of amenities in fostering urban consumption. We collected over 120,000 comments spanning 60 months (2015–2019) from 18 urban shopping centers in Shanghai. By applying text mining methods, we constructed a panel data model on the perception of four types of amenities and consumer emotions. Results indicate that different perceptions of amenities yield significantly different effects on consumer emotions. Specifically, we found that perceptions of cultural and safeguarded amenities significantly influence consumer emotions, albeit with different directions of impact. In contrast, perceptions of commercial and natural amenities did not significantly affect consumer emotions. The findings of this study provide a key reference for how to scientifically plan and reasonably introduce the types of amenities in urban consumption space, so as to reflect the promoting effect of amenities on urban consumption.

## 1 Introduction

With the ongoing trend of centralization in consumption, urban areas have significantly improved the quality of life by providing convenient urban consumption spaces. These spaces introduce a variety of amenities, such as museums, art galleries, libraries, cinemas, bars, restaurants, and farms, offering commercial and cultural services that make city residents feel comfortable and happy. Combining these amenities transforms urban consumption spaces into vital channels for commercial transactions and access to public services. Moreover, they become indispensable emotional havens in the daily lives of urban residents.

The concept of ’amenities’ originally stems from economics and is closely related to consumption. Amenities represent the consumption function within cities, and the interaction between urban space and citizen-consumers forms the core of amenity theory [1]. However, domestic and international scholars have tended to overlook the micro-emotional factors underlying amenities’ role in promoting consumption. Academic research on amenities has predominantly focused on macro-level themes, such as their impact on urban development and population migration. While most scholars agree that amenities can evoke pleasurable emotions in consumers, only a few studies have quantitatively demonstrated this phenomenon. Therefore, there remains a need to explore the distinct effects of various amenity types on consumer emotions.

Amidst the evolving landscape of urban consumption spaces, amenities have emerged as crucial factors in attracting consumers. To effectively stimulate positive consumer emotions and enhance overall satisfaction, it is imperative to introduce amenities that align with current consumption trends thoughtfully. So that we can underscore the pivotal role of amenities in promoting urban consumption. In this context, leveraging big data text mining and panel data analysis, this study addresses the limitations of traditional questionnaire survey methods in capturing consumer emotions. By evaluating the texts from 18 urban shopping centers in Shanghai over 60 months (2015–2019), this study explores the impact of four types of perceptions of amenities on consumer emotions in the urban consumption space. This research provides invaluable insights for scientifically planning and sensibly introducing various amenity types in urban consumption spaces. By understanding the correlation between these amenities and consumer emotions, businesses and policymakers can make informed decisions to create more appealing and satisfying environments for urban consumers.

## 2 Theoretical background

### 2.1 Study of consumer emotions

There needs to be a unified understanding of the definition of consumer emotions in academic circles. However, in general, we think that consumer emotions encompass consumers’ reactions when purchasing a product or experiencing a service. Numerous studies have investigated the relationship between consumer emotions and consumer satisfaction, consistently finding that consumer emotions significantly predict consumer satisfaction [2]. In most cases, a positive relationship exists between consumer emotions and satisfaction [3]. Consequently, consumer emotion measures also indicate consumer satisfaction with urban consumption spaces. Furthermore, several studies have explored the factors influencing consumer emotions, with a particular focus on consumer pre-emotions [4], service staff’s behaviors and attitudes [5], service environment [6], and service quality [7]. The service environment comprises two categories: service scenes and physical facilities, which are closely related to amenities.

In terms of service scenes, extensive and scattered research suggests that factors such as lighting, air quality, temperature, music, and scent have the potential to significantly influence consumer emotions [8–10]. Bigdeli et al. argue that atmospheric elements such as ambiance, interaction, and design affect customer emotions [11]. Additionally, Teller et al. [12] contend that environmental factors, including a consumer space’s layout, pleasantness, and overall atmosphere, can greatly affect its overall attractiveness.

Regarding physical facilities, So et al. [13] argue that an exceptional physical environment fosters positive consumer emotions. Ali and Amin emphasize that customers with higher perceptions of the physical facilities are more likely to experience positive emotions [14]. In a study related to hotels, Wang et al. [15] found that consumers’ positive emotions predominantly arise from the quality of service facilities. Even in online hotel comments, consumers tend to emphasize service facilities more [16], and the overall rating of hotels is primarily contingent on consumers’ satisfaction with the physical facilities [17–19].

The service scenes and physical facilities these studies focus on are distinct from amenities. The research scope mainly centers on service consumption spaces, such as restaurants and hotels, with only a few researchers investigating broader and larger-scale consumption spaces, such as urban shopping centers.

### 2.2 The relationship between amenities and emotions

Amenities are not merely businesses or facilities; they play a crucial role in fostering emotional exchange and cultural cohesion among residents. They serve as expressions of individuals’ aspirations for a better life while carrying the local memories and the inheritance of urban culture. Given the relatively complex emotional experience associated with amenities, we aim to elucidate their relationship with emotions through three key aspects: a sense of pleasure, comfort, and place.

#### 2.2.1 Pursuit of individual-centered pleasure

Amenities revolve around people, and they exist and evolve with a strong anthropocentric orientation [20]. Ultimately, the most fundamental human need is to experience pleasure. The correlation between amenities and pleasure is evident from the definition of amenities. For instance, Wang Ning posits that amenities refer to objective things, states, or environments that provide people with a sense of comfort and pleasure [21]. Silver, Clark, and their colleagues at the Chicago Urban School [22] define amenities as the pleasure derived from the use or enjoyment of goods and services.

The development of amenities has always revolved around a specific orientation towards pleasure and has exhibited a progression of pleasure levels. Before the extensive commercialization, amenities mainly focused on public properties, offering the most basic level of pleasure. For instance, the early emergence of churches and temples symbolized religious gratification. In contrast, the later emergence of museums and libraries represented humanistic enjoyment, while the development of cafes and online celebrity restaurants embodied the pleasure of modern consumption. As entities that bring joy and satisfaction to people, amenities are continuously evolving to meet the elevated pleasure needs of individuals.

#### 2.2.2 Comfort from high quality of life

Numerous scholars have argued that one of the crucial roles of amenities is to improve the quality of life. A central reason for this assertion is that amenities are precisely what render certain places attractive for both living and working [23]. Brambilla further argues that elevating the level of amenities directly correlates with an improvement in quality of life [24]. Additionally, Albouy contends that amenities play a decisive role in determining the overall quality of life within a given area [25]. The utilization of amenities by human beings reflects their aesthetics and the pursuit of a comfortable life. Clark points out that amenities contain the attitudes and values of residents [26]. This pursuit of a high quality of life involves emotional elements and serves as a yardstick for understanding the emotional tendencies generated.

The eternal quest for a high quality of life is the driving force behind the existence of cities. The essence of quality of life lies in the ability to possess and enjoy material goods and services that bring pleasure and comfort [27]. Gordillo remarked that people build cities to cultivate urban life [28]. The concept of amenities marks a transition in urban form from a production-based society to a consumption-based society. Achieving a high quality of life is, therefore, contingent upon consumption. By utilizing city-provided amenities, urban dwellers attain and experience the essence of urban life. As a result, they benefit from a heightened quality of life characterized by feelings of comfort, pleasure, and fulfillment.

#### 2.2.3 Sense of place as an urban attraction

The primary aim of amenities in improving the quality of life is to benefit the local residents, as they are highly regional and cannot be mass-produced. The amenity richness theory principle pillar is the local place-based characteristics that satisfy the quality of life [29]. According to Silver et al. [30], local residents express shared emotions through the experience and consumption of amenities, fostering interactions between them, promoting a familiar and intimate emotional attachment to the place, and consequently attracting people to gather and form micro-communities or even entire cities invisibly. As a result, some scholars believe that cities rich in amenities will attract more people to migrate [31]. However, the quantity of amenities serves only as an explicit indicator of a city’s attractiveness. The implicit indicator, in contrast, lies in the emotional connection that amenities form with the place, evident in people’s daily lives. Maling [32] argues that amenities transform a city from a mere hub of economic growth to one that prioritizes happiness. Scott [33] contends that the appeal of amenities lies in the spiritual satisfaction people derive from their environment. Thus, the cultivation of a sense of place by amenities becomes an essential factor in enhancing the attractiveness of cities.

### 2.3 Study of the classification of amenities and their impact on the sense of well-being

There is no standardized classification of amenities in the academic world. The categorization of amenities is an ever-evolving process, adapting to the changing content of people’s needs. For instance, Clark [34] identified four main categories of amenities: natural physical amenities, constructed amenities, socio-economic composition and diversity, and values and attitudes of residents. Glaeser [35], conversely, classified amenities into four groups: a wide variety of services and consumer goods, aesthetic and physical settings, good public services, and speed. Chinese scholars have reclassified amenities in the context of China’s livable city indicator system and national conditions. For instance, Wang Ning [36] categorized amenities into six groups: natural environment, built environment, commercial life, public goods, infrastructure, and social atmosphere. Likewise, Ma Ling et al. [37] divided amenities into six categories: natural, cultural, commercial, transportation, health, and social.

Overall, the academic classification of amenities has remained relatively stable for some time. Based on the existing categorization, we examine the development of urban consumption space in China, focusing on the cultural and commercial amenities that dominate this sphere. Transportation, health, and social amenities, which constitute a relatively small proportion, are categorized as safeguarded amenities. Considering the emergence of natural amenities as a new type of urban consumption space in recent years, they are also included in the classification. Consequently, the amenities are classified into four distinct categories: cultural amenities, commercial amenities, safeguarded amenities, and natural amenities. This standardized amenity classification will be utilized consistently throughout the empirical tests in the subsequent sections.

A need for more research directly explores the four types of amenities and their impact on consumer emotions, as most studies tend to concentrate on the effects of various amenity types on the sense of well-being. Specifically.

The first type of cultural amenities comprises the primary places for people’s cultural practices and recreation, such as cultural and art venues, performing arts and amusement centers, movie theaters, bookstores, cafes, and more. Urban residents highly value the spiritual significance of cultural amenities [38]. Cultural amenities enhance people’s quality of life [39], and high quality of life also significantly contributes to accelerating subjective well-being [40]. Numerous scholars have explored the relationship between cultural amenities and subjective well-being, with most agreeing that cultural amenities positively impact subjective well-being [41]. For instance, Hong et al. [42] argued that cultural opportunities, including leisure activities and access to cultural facilities, positively influence subjective well-being.

The second category of commercial amenities comprises essential social consumer goods closely related to urban life, including restaurants, clothing stores, supermarkets, and other facilities. These commercial amenities address urban residents’ most fundamental survival needs and set the minimum standard for human well-being. As urban commerce continues to thrive, the variety of commodities available grows by the day, providing individuals with a broader range of choices, ultimately leading to increased happiness among people. Consequently, the development of commercial amenities is directly linked to the well-being levels of urban residents.

The third category of safeguarded amenities mainly pertains to the degree of social inclusion, cultural literacy of the residents, spiritual civilization, and the overall safety of the locality, such as transportation convenience, medical facilities, leisure options, and security infrastructure. These safeguarded amenities play a crucial role in safeguarding individuals’ fundamental rights to survival and development. They also help improve unfavorable conditions in society, culture, and nature that affect human life, ultimately enhancing individual freedoms [43]. As a result, safeguarded amenities are considered one of the primary sources of residents’ sense of well-being. Scholars have verified the significant positive impact of safeguarded amenities on residents’ sense of well-being [44].

The fourth category of natural amenities mainly refers to livable natural conditions, such as climate and air quality. Natural amenities are created due to the scarcity of natural resources in the process of industrialization and urbanization, catering to the realistic needs of human beings to be close to nature. People’s preference for natural amenities has recreational and aesthetic values, and the existence of such amenities reflects human well-being. Numerous scholars have pointed out that climatic factors have a significant impact on the sense of well-being [45, 46]. Green spaces in cities contribute to the subjective well-being of residents [47, 48], while poorer air quality increases anxiety [49].

There exists a positive relationship between four categories of amenities and a sense of well-being. Furthermore, a sense of well-being is a manifestation of positive emotions [50]. Field [51] asserts that happiness involves doing one’s best to make others happy. Haybron [52] proposes that we regularly use happiness as a proxy for well-being. Diener [53] believes subjective well-being comprises emotional experiences and life satisfaction. Thus, building upon the aforementioned theoretical foundations, we present the following hypotheses.

**H1.** The perception of amenities plays a significant positive role in influencing consumer emotions.

**H1A. T**he perception of cultural amenities significantly and positively affects consumer emotions.

**H1B.** The perception of commercial amenities significantly and positively affects consumer emotions.

**H1C.** The perception of safeguarded amenities significantly and positively affects consumer emotions.

**H1D.** The perception of natural amenities significantly and positively affects consumer emotions.

## 3 Research design

### 3.1 Model

To examine the impact of amenities on consumer emotions, we utilize a panel data approach for empirical analysis (S1 Dataset). The big data is derived from evaluating 18 urban shopping centers in Shanghai over 60 months from 2015 to 2019. We control for change factors at individual and time levels to address potential endogeneity issues. Accordingly, we construct a two-way fixed effects model that incorporates both time and individual effects as follows:

$${Emotion}_{it}=\alpha_{0}+\alpha_{1}{Amenity}_{it}+\alpha_{2}{Place}_{it}+\rho_{i}+\tau_{t}+\varepsilon_{it},$$

where the subscripts i and t denote individual and month, respectively, Emotion_it_ denotes the explanatory variable; Amenity_it_ is the core explanatory variable for the perception of amenities; while Place_it_ serves as the control variable for the perception of place; ρ_i_ and τ_t_ indicate the individual and month fixed effects, respectively; and ε_it_ represents the random perturbation term.

### 3.2 Data gathering

The specific form of Chinese urban consumption space primarily comprises department stores or urban shopping centers located in city centers. These urban consumer spaces have evolved into popular gathering places due to their vibrant consumer atmosphere and well-established sales chains. As one of the cities with the highest consumption levels and the best consumption environment in China, Shanghai has witnessed a rapid transformation and upgrade of its urban consumption space in recent years. This development has been driven by the strategic goal of establishing an international consumption city. Consequently, the number of amenities in urban shopping centers has increased significantly, and the speed of iterative updating has accelerated. Therefore, we select Shanghai’s urban shopping centers as the primary data collection subject.

Due to the substantial number of comments and comprehensive representation of urban shopping centers available on WWW.DIANPING.COM, we focus on data collection through this platform. Considering the potential impact of the COVID-19 outbreak in China in 2020 on data reliability, the data collection period is set from 2015 to 2019, spanning five years. The data is sorted based on three key indicators: activity, per capita consumption, and comment volume, in descending order. To be included, the comments must span at least five years (except for New World Daimaru Department Store, which had one month of missing data that we compensated for using the mean method). Ultimately, 18 urban shopping centers were chosen for analysis (Table 1). We collected 127,966 evaluation texts, which serve as the primary data source for constructing our study.

**Table 1:** List of data collection objects and volume of comments (2015–2019).

Serial number	Shopping center name	Earliest comment time	Number of comments	URL
1	Jiuguang Department Store	2006/4/11	5253	https://www.dianping.com/shop/k4KvrKKhufWmsftW
2	Longemont Shopping Mall	2006/4/10	8737	https://www.dianping.com/shop/H54yvcJBQCV3qfIQ
3	Super Brand Mall	2006/4/19	5993	https://www.dianping.com/shop/ikS4HrkIMtYNA4dS
4	Shanghai Raffles City	2006/4/11	6353	https://www.dianping.com/shop/l2zkldsTt3JRVeoB
5	Grand Gateway	2006/4/14	5161	https://www.dianping.com/shop/l4vOuItwfzr5cR76
6	Metro City	2006/6/27	5601	https://www.dianping.com/shop/G8HjViUMLA7HHWmt
7	Life Hub Daning	2006/9/20	3496	https://www.dianping.com/shop/FjStkvIw2je93mMn
8	Shanghai K11 Art Mall	2009/8/5	7113	https://www.dianping.com/shop/k7t6XuznEZruHNv2
9	Sun Moon Light Cente17r	2010/4/14	8706	https://www.dianping.com/shop/l4zIgBycAXtVNR4U
10	Shanghai Jingan Joycity	2010/12/2	20905	https://www.dianping.com/shop/G5wcnV4FRCryz6vb
11	Hong Kou Plaza	2011/1/12	5651	https://www.dianping.com/shop/H5gEU5YquerWml2u
12	IAPM Mall	2011/10/18	8637	https://www.dianping.com/shop/l28MGCyy5YW9Rd2V
13	The River Mall	2012/12/28	6803	https://www.dianping.com/shop/iGVlukyPv84ijILf
14	IFC Mall	2013/1/13	5516	https://www.dianping.com/shop/H78O9Px8UITvFrj6
15	Jing An Kerry Center	2013/8/31	4212	https://www.dianping.com/shop/kaeSEj3gWQw0joBS
16	Changtime Plaza	2014/9/13	5014	https://www.dianping.com/shop/FybJwTCG7cVWNO8b
17	The Place	2014/9/13	7945	https://www.dianping.com/shop/l4j0WiEQQPNCBAKG
18	Shanghai New World Daimaru Department Store	2015/2/2	6870	https://www.dianping.com/shop/H8GlKVnm0ZdxoKw9
Total			127966	

### 3.3 Variable setting

Based on the research hypotheses, six variables were constructed. These variables include the explanatory variable of consumer emotions, the explained variable of perceptions of the four types of amenities, and the control variable, the perception of place. We applied a data preprocessing step to ensure the accuracy of the estimation results and prevent the influence of outliers in the model. Specifically, we shrunk the tails of all continuous variables from 1% to 99% before conducting testing and regression. Table 2 presents all variables’ meanings and descriptive statistics after the shrinkage process.

**Table 2:** Descriptive analysis of variables (N = 1080 n = 18 T = 60).

Variable name	Variable symbol	Between/within	Group mean	Standard deviation	Minimum	Maximum
Consumer emotions	Emotion	overall	0.809	0.0747	0.388	0.991
		between		0.0351	0.740	0.871
		within		0.0664	0.432	0.972
Perception of cultural amenities	Culture	overall	0.237	0.167	0	1
		between		0.126	0.0374	0.578
		within		0.114	-0.196	0.847
Perception of commercial amenities	Commerce	overall	0.234	0.126	0	0.792
		between		0.0834	0.0949	0.477
		within		0.0966	-0.0709	0.617
Perception of safeguarded amenities	Safeguard	overall	0.151	0.0999	0	0.565
		between		0.0755	0.0713	0.414
		within		0.0678	-0.106	0.529
Perception of natural amenities	Nature	overall	0.0682	0.0745	0	0.341
		between		0.0600	0.0202	0.252
		within		0.0463	-0.121	0.351
Perception of place	Place	overall	0.334	0.167	0.0294	0.848
		between		0.116	0.124	0.645
		within		0.123	-0.132	0.995

#### 3.3.1 Explained variable: Consumer emotions

Numerous studies have been conducted in the academic community on developing consumer emotion scales. Consumer emotions are generally regarded as continuous, ranging from pleasant to unpleasant [54], or they can be categorized as positive and negative emotions [55]. However, distinguishing between positive and negative consumer emotions during actual consumption experiences often proves challenging [56]. Therefore, we adopt the first viewpoint, considering consumer emotions as a continuous process, and utilize the SnowNLP emotion dictionary in the Python class library to mine the emotional content of network evaluation texts. This approach allows us to obtain an emotion value that varies within the 0 to 1 interval. It is essential to acknowledge that consumer emotions constantly change, and pre-emotions can influence post-emotions. Consequently, cross-sectional research methods may need to analyze real consumer emotions objectively. To address this limitation, we advocate tracking data over an extended period to measure consumer emotions better. We base our analysis on the above emotional values and perform a summed average calculation. By constructing the explained variable ’consumer emotions’ at a monthly granularity, we aim to offer a more comprehensive understanding of consumer emotions.

#### 3.3.2 Explanatory variables: Perception of amenities

We present the construction of perception of amenities using a topic mining method applied to comment text. Firstly, we consider all comments a collective entity and extract implicit themes from the text using the LDA topic model and TF-IDF algorithm. After applying the TF-IDF method and determining the weights of each keyword, we incorporate additional terms like ambiguous words, neologisms, and network words into a customized dictionary based on the weight-ordering results. Subsequently, we utilize the Jieba library in Python, the adjusted customized dictionary, and the thesaurus for the HIT shutdown to redivide the words and calculate word frequencies. We select 418 amenities-related keywords that occur more than 100 times from the resulting data. By combining and replacing similar keywords, we consolidate amenities in each comment and treat multiple occurrences of synonymous keywords as one. To classify the keywords, we establish four primary categories representing the perception of different types of amenities: cultural amenities, commercial amenities, safeguarded amenities, and natural amenities (Table 3).

**Table 3:** Keyword classification framework.

Core Variables	Subcategories
Perception Of Cultural Amenities	Exhibition or Pavilion, Performance, Amusement, Cinema, Bookstores, Drinks Shop
Perception of Commercial Amenities	Supermarket, Clothing Store, Cosmetic Store, Restaurant, Experience Store
Perception of Safeguarded Amenities	Parking, Hygiene, Inquiries, Rest, Health
Perception of Natural Amenities	Flora and Fauna, Artefacts, Lights And Shadows
Perception of Place	Transportation Routes, City Roads, Landmarks

Note: Due to the limited space, this article only lists the keyword classification framework, the specific keyword list is shown in S1 Table.

Finally, considering the variations in the number of comments for shopping centers in each city, we normalize the total frequency of keywords within each category by dividing it by the number of comments. This process yields the number of mentions per capita for each amenities category. Then we use this data to construct panel data for the four significant categories of perceptions of amenities, taking into account monthly granularity.

#### 3.3.3 Control variable: Perception of place

It is common for people to develop an emotional connection to familiar places [57]. Public spaces and landmarks with bustling social activities are the primary locations where emotional attachments are formed. Notably, these places also serve as the main hubs for shopping centers in Chinese cities. The emotions expressed by consumers in the text may partly stem from their perception of these distinctive places. We identified three types of place-related keywords from the keywords that appeared more than 100 times: public transportation routes, urban roads, and landmark architectural places (Table 3). To construct the perception of place variable, we followed the method used for constructing the perception of amenities variable and included it as a control variable in the model.

## 4 Results of empirical analysis

### 4.1 Cross-sectional correlation test

Pesaran [58] demonstrated that the first-generation panel unit root test is only reliable when dealing with cross-sectional uncorrelation. Hence, conducting a cross-sectional correlation test before employing the unit root test is essential. If a cross-sectional correlation exists, the appropriate choice is to use the second generation of the panel unit root test. The cross-sectional correlation test comprises several methods, including the LM test proposed by Breusch and Pagan [59], the Pesaran CD test proposed by Pesaran [58], the Pesaran scale LM test proposed by Pesaran et al. [60], and the Bias-scale LM test proposed by Baltagi et al. [61]. We consider these four methods, which are commonly used, to ensure the robustness of the tests.

As presented in Table 4, the CD test for the perception of safeguarded amenities is the only variable that shows significance at the 10% level. All six variables exhibit significance at the 1% level, leading us to reject the original hypothesis that there is no cross-sectional correlation in the panel data. Consequently, a more pronounced cross-sectional correlation exists in the perception of amenities across all 18 shopping centers. This observation suggests that the data in this study, derived from the same geographic region and industry, may be influenced by a common shock affecting all individuals, thereby leading to the panel data cross-sectional correlation.

**Table 4:** Results of cross-section correlation test.

Test	Emotion	Culture	Commerce	Safeguard	Nature	Place
Breusch-Pagan LM	806.429***	437.390***	461.270***	285.151***	214.310***	1409.168***
Pesaran scaled LM	37.354***	16.257***	17.623***	7.555***	3.505***	71.810***
Bias-corrected scaled LM	37.201***	16.105***	17.470***	7.402***	3.352***	71.658***
Pesaran CD	22.128***	12.513***	16.295***	1.688*	2.976***	32.152***

*** indicates significance at the 1% level

** indicates significance at the 5% level, and

* indicates significance at the 10% level.

### 4.2 Unit root test

Due to the extended period and the nature of the panel data selected for this study (characterized as "big T, small N"), it is crucial to avoid pseudo-regression situations. To achieve this, we employ unit-root tests to assess the data. However, it is worth noting that the first generation of panel unit root tests may introduce bias in the test statistic due to cross-section correlation. We adopt the second-generation unit root test programs to address the problem of biased estimates, namely the Panicca and CIPS tests, designed to handle cross-section correlation effectively. Table 5 presents the results of these two tests. Notably, all variables strongly rejected the original hypothesis of the presence of a unit root. This outcome indicates that the panel series in our study exhibits smooth behavior, meeting the necessary smoothness requirements for accurate modeling and regression analysis.

**Table 5:** Unit root test results.

Variable	Pannica test statistic			CIPS*test
	P_a_	P_b_	PMSB	Statistic
Emotion	-23.492***	-7.83***	-2.524***	-5.781***
Culture	-19.433***	-6.089***	-1.993**	-5.585***
Commerce	-49.224***	-11.53***	-2.535***	-5.898***
Safeguard	-59.187***	-13.15***	-2.774***	-5.434***
Nature	-34.666***	-9.305***	-2.446***	-5.886***
Place	-14.81***	-4.902***	-1.748**	-5.802***

*** indicates significance at the 1% level

** indicates significance at the 5% level, and

* indicates significance at the 10% level.

### 4.3 Results of the benchmarking regression

We employ a two-way fixed effects model to examine the impact of four types of amenities on consumer emotions. The results from Models (1) to (4) presented in Table 6 indicate that the perceptions of cultural, commercial, and safeguarded amenities all have a statistically significant effect at the 1% level. Moreover, the perception of natural amenities substantially affects the 5% level. The findings demonstrate that all four types of perceived amenities significantly influence consumer emotions, which aligns well with the hypotheses of this study. However, this result also shows that the perceptions of cultural, commercial, and natural amenities positively affected consumer emotions, while perceptions of safeguarded amenities negatively impacted consumer emotions. These results suggest that the higher consumers’ perceptions of cultural, commercial, and natural amenities, the more positively they tend to feel about urban consumption spaces.

**Table 6:** Two-way fixed effects model regression.

Variable	Emotion			
	(1)	(2)	(3)	(4)
Culture	0.111***			
	(0.018)			
Commerce		0.077***		
		(0.024)		
Safeguard			-0.137***	
			(0.032)	
Nature				0.103**
				(0.050)
Place	0.100***	0.101***	0.126***	0.105***
	(0.020)	(0.021)	(0.021)	(0.021)
_cons	0.749***	0.757***	0.787***	0.767***
	(0.008)	(0.009)	(0.008)	(0.008)
N	1080	1080	1080	1080

*** denotes significance at the 1% level

** denotes significance at the 5% level, and

* denotes significance at the 10% level.Standard errors are in parentheses.

Conversely, a higher perception of safeguarded amenities leads to more negative emotions regarding the urban consumption space. The observed deviation in the influence of perceived safeguarded amenities on consumer emotions from theoretical expectations calls for further analysis in future research.

Furthermore, the study controlled for the effect of the perception of place on consumer emotions across all four models. The empirical results demonstrate that the impact of perception of place on consumer emotions is consistently positive and statistically significant at the 1% level. This result suggests that consumer attention toward specific places significantly fosters positive consumer emotions.

### 4.4 Endogenous problems

The model may suffer from endogeneity issues, leading to contradictory regression coefficients for individual amenities on consumer emotions. We address the problem by re-estimating the model using a two-stage least squares approach. To mitigate instrumental variable selection bias, we conservatively select the first-order lag terms of perceptions of various types of amenities as instrumental variables. Additionally, we employ robust standard errors for the parameters to account for heteroskedasticity. The results of models (5)-(8) in Table 7 demonstrate that perceptions of cultural and safeguarded amenities significantly impact consumer emotions at the 1% significance level. In contrast, the perceptions of commercial and natural amenities no longer maintain their original significance, and the direction of the regression coefficients also changes. This difference in results, as presented by models (6) and (8), effectively confirms the presence of an endogeneity problem in the original model. After controlling for endogeneity, the effect of perceptions of cultural and safeguarded amenities on consumer emotions remains consistent in direction. However, after accounting for endogeneity, the regression coefficient becomes more pronounced, indicating a stronger effect of amenities on consumer emotions. The significance and direction of the control variables remain unchanged. It is worth noting that all instrumental variables pass both the weak instrumental variable test and the under-identification test.

**Table 7:** Endogeneity tests for two-way fixed effects models.

Variable	Emotion			
	(5)	(6)	(7)	(8)
Culture	0.191***			
	(0.044)			
Commerce		-0.070		
		(0.227)		
Safeguard			-0.348***	
			(0.126)	
Nature				-0.149
				(0.311)
Place	0.094***	0.115***	0.155***	0.113***
	(0.020)	(0.029)	(0.026)	(0.024)
N	1062	1062	1062	1062
Weak identification test	172.23***	11.336***	68.553***	23.378***
Underidentification test	55.717***	7.56***	44.637***	10.756***

*** denotes significance at the 1% level

** denotes significance at the 5% level, and

* denotes significance at the 10% level.Standard errors are in parentheses.

Combining the results of the above models leads to the following conclusions: the perception of cultural amenities has a significant positive influence on consumer emotions, thereby verifying hypothesis H1A. However, the influence of perceptions of commercial and natural amenities on consumer emotions is insignificant after controlling for endogeneity. As a result, hypotheses H1B and H1D fail to be verified.

### 4.5 Other robustness tests

To further validate the robustness of the regression results, we reconstructed and regressed the explanatory variables. The original explanatory and control variables’ values were incremented by one and then logged to generate four new sets of amenity variables and place variables. The study included the above variables in a two-way stationary model. Subsequently, we again conducted the same endogeneity test to derive the Table 8 models (9)-(12).

**Table 8:** Robustness tests for two-way fixed effects models.

Variable	Emotion			
	(9)	(10)	(11)	(12)
LnCulture	0.258***			
	(0.068)			
LnCommerce		-0.092		
		(0.298)		
LnSafeguard			-0.409***	
			(0.148)	
LnNature				-0.224
				(0.399)
LnPlace	0.119***	0.155***	0.208***	0.155***
	(0.028)	(0.041)	(0.036)	(0.034)
N	1062	1062	1062	1062
Weak identification test	130.531***	10.833**	69.439***	16.615***
Underidentification test	55.111***	7.702***	45.301***	7.714***

*** denotes significance at the 1% level

** denotes significance at the 5% level, and

* denotes significance at the 10% level.Standard errors are in parentheses.

The results demonstrate that the direction of the coefficients and the significance level of the effect of each type of perception of amenities on consumer emotions have mostly stayed the same. This result reaffirms the robustness of the previous model, further reinforcing its reliability.

## 5 Conclusions and discussion

### 5.1 Conclusions

This study has presented a comprehensive analysis of more than 120,000 cumulative comments of 18 urban shopping centers in Shanghai, covering a period of 60 months from 2015 to 2019, obtained from WWW.DIANPING.COM. The primary objective was to construct panel data focusing on four central perceptions of amenities and consumer emotions. We have adopted a two-factor fixed-effects model and an endogeneity test to explore the impact of various types of amenities on consumer emotions. Our findings are as follows:

The impact of perceptions regarding amenities on consumer emotions varies significantly depending on the types of amenities. Specifically, perceptions of cultural and safeguarded amenities significantly affect consumer emotions, but they influence them in different ways.

When consumers perceive cultural amenities more strongly, their emotions become more positive. Previous research has predominantly focused on the positive impact of cultural amenities on well-being [62]. Since consumer emotion is a component of well-being, this result is generally consistent with prior research. In our specific comment, we also observed that cultural amenities have indeed become the main attraction of urban shopping centers. iiMedia Research data shows that cultural, sports, and entertainment formats in shopping centers opened in China in 2018 accounted for the highest proportion of all formats, reaching 38% [63]. The role of cultural amenities in influencing consumer emotions is one of the main reasons why urban shopping centers can build their attraction and differentiate themselves from the competition.

While perceptions of safeguarded amenities also significantly influence consumer emotions, stronger perceptions of safeguarded amenities tend to evoke more negative emotions in consumers towards urban shopping centers. Several studies have shown that in many public spaces, safeguarded amenities provide consumers with a sense of safety, thereby eliciting positive emotions [64]. While this study demonstrates that safeguarded amenities are associated with personal mental states and emotions, it reveals an inverse influence effect. A closer examination of comments mentioning safeguarded amenities reveals that the majority of consumers consider them as necessary basic amenities in urban shopping centers. Some consumers view them as essential points of information and express objective and calm sentiments in their comments, while others are quite critical of safeguarded amenities, particularly concerning parking-related facilities. This may account for the negative impact of safeguarded amenities on consumer emotions.

In the initial model, commercial and natural amenities significantly impact consumer emotions. However, upon conducting the endogeneity test, the result revealed that these amenities did not significantly affect consumer emotions. In previous studies, we have been more inclined to believe that commercial amenities have a positive effect on consumer emotions. Das et al. suggest that a large number of retail stores must be installed in urban shopping centers due to the positive correlation between spatial congestion and consumer emotions in shopping centers [65]. These retail stores, forming the commercial amenities, provide convenience and a variety of products, making them the most crucial component of urban shopping centers. However, as urban shopping centers are perceived as conglomerates of planned retail formats [66], consumers have become accustomed to their presence and are less likely to write new comments about them. Moreover, with the rise of online retailing, the number of Chinese consumers shopping online has exceeded 900 million [67], altering Chinese consumers’ reliance on offline commercial amenities. All of these factors may contribute to a lower level of consumer perception of commercial amenities in urban shopping centers.

It has been demonstrated that urban shopping centers can enhance customers’ spirituality, health, or well-being through green elements or green spaces [68]. One possible reason for this is that in previous studies, natural amenities referred only to greenery used for interior decoration. However, with the rising demand for consumer experience, more natural amenities beyond greenery are being introduced into urban shopping centers. Therefore, this paper significantly broadens the range of natural amenities when constructing keywords. In addition to green plants, this paper includes artifacts, light and shadow effects, and small animals. These newly included types of amenities may occupy a small scale in urban shopping centers and may not attract much attention from consumers. Another reason for the observed differences may be that almost all of the Shanghai shopping centers in this study are enclosed, whereas previous studies have focused on the construction of natural spaces outside shopping centers [69]. The differences in the design styles of shopping centers in different countries may have contributed to these results.

As a result, consumers’ perceptions of both types of amenities, namely commercial amenities and natural amenities, had an insignificant impact on their emotions. This conclusion remains consistent even after conducting the robustness test. Hence, among the four types of amenities studied, cultural and safeguarded amenities emerge as the most crucial factors in enhancing positive emotions among consumers in urban consumption spaces.

### 5.2 Managerial implications

Based on the conclusions presented in this study, we derive the following policy insights from the current state of urban consumption space development in China:

Firstly, we should incorporate cultural amenities to enhance consumer emotions in the urban consumption space. With the rising demand for culture and leisure among urban residents, these spaces have become central hubs for cultural and recreational activities. Spiritual consumption has taken center stage in urban consumption spaces. Therefore, we must integrate cultural amenities that cater to spiritual consumption, staying abreast of the latest cultural and leisure consumption trends. Moreover, we also need to facilitate transforming and modernizing traditional urban consumption spaces while leveraging cultural amenities to elevate consumer satisfaction and foster consumer loyalty.

Secondly, we should strive to improve and enhance software and hardware quality related to safeguarded amenities in the urban consumption space. Safeguarded amenities are vital facilities in the urban consumption space that ensure the physical comfort of consumers, and they are also highly susceptible to consumer criticism. To address this, we should closely monitor consumer feedback regarding safeguarded amenities and develop strategies to elevate the provision of such amenities while enhancing the service levels of parking and sanitary facilities. Additionally, enhancing digital management, improving accessibility to public transportation, and fostering collaboration between government, shopping center managers, and community residents will collectively create a welcoming and comfortable space, ultimately leading to improved consumer satisfaction.

Thirdly, we should thoughtfully adjust the layout of amenities to enhance and increase the proportion of natural elements in the urban consumption space. Depending on the location of the urban consumption space and the characteristics of the consumer group, we can implement a well-planned distribution of various types of amenities, carefully considering the introduction of natural amenities to complement any shortcomings and ensure a harmonious balance with other amenities. Emphasis should be placed on the development and scale of natural amenities, harnessing their potential to transform spaces and create new consumption areas that enrich consumers’ connection with nature.

### 5.3 Limitations and suggestions for further research

Although we have reached some conclusions, the underlying reasons behind this conclusion require further research. The data source of this study is limited to a single city, Shanghai, and conducting a multi-city or cross-dimensional comparative study could provide valuable insights in the future. Furthermore, from a fieldwork perspective, subsequent studies should focus on qualitatively analyzing the reasons for the reverse effect of safeguarded amenities and the non-significant effects of natural and commercial amenities.

## Supporting information

S1 Table Keyword list. This file provides 418 amenities-related keywords.
(DOCX)

S1 Dataset Panel data on perceptions of four types of amenities in 18 urban shopping centers in Shanghai, 2015–2019 (60 months in total). (XLS)
