# Supplementary material for: Influence of the perceptions of amenities on consumer emotions in urban consumption spaces
Source: PLoS One. 2024 May 29;19(5):e0304203. doi: 10.1371/journal.pone.0304203 (PMC11135775; doi:10.1371/journal.pone.0304203)
Supplement: S1 Table — This file provides 418 amenities-related keywords. (DOCX) [file pone.0304203.s001.docx]

**Keyword List**

| **Core Variables** | **Subcategories** | **Similar Keywords** |
| --- | --- | --- |
| **Perception Of Cultural Amenities** | **Exhibition or Pavilion** | Exhibition, Museum, Exhibition Hall, Exhibition Pavilion, Exhibition Space, Fair, Convention, Special Exhibition, Garfield, Kitty, Line, Lego, Ultraman, Cherry Bomber, Bear, Tom Bear, Teddy Bear, Spongebob Squarepants, King Of Thieves, Minute Maid Warrior, Pikachu, Gundam, Conan, Snoopy, Balloon, Gudeta, Saint Seiya, Doraemon, Toy Story, Disney, Mickey, Panda, Hoodie, Frozen, Magic Beasts, Traveling Frogs, The Little Prince, Qiaohu, Jay Chou, Art Exhibition, Painting Exhibition, Dali, Gaudi, Monet, The Forbidden City, Wooden Structure, Museum Of Fine Arts, Art Gallery, Sculpture, Statue |
|  | **Performance** | Acting, Magic, Circus, Theater, Opera, Stage, Performance, Drama, Crush, Band, Concert, Meet & Greet, Roadshow |
|  | **Amusement** | Pleasure Ground, Amusement Parks, Touring Parties, Carnivals, Theme Parks, Amazon, Kiddie Parks, Bubble Mart, Popmart, Amusement, Video Games, Games, Haunted Houses, Slides, Mazes, Big Heads, Twister, Crane Machines, Trains, Thomas, Carousels, Swings, Ferris Wheels, Carousels, Sky Ring, Skating Rinks |
|  | **Cinema** | Cinema, Movie Theatre, Cinema City, Palace, Movie-Plex, Ktv, Holliday, Silver Lodi |
|  | **Bookstores** | Bookstores, Popular Holdings, Book Signings |
|  | **Drinks Shop** | Coffee Shop, Coffee Palace, Café, Coffee Bar, Coffee House, Cafe, Costa, Pi Master, Manner, Stayreal, Drinks Shop, Milk Tea Shop, Lelouch Tea, Bar, Drinks Shop, Milk Tea Shop |
| **Perception of Commercial Amenities** | **Supermarket** | Supermarket, Pokphand Lotus, Carrefour, Rt-Mart, Lotus, Freshhema, Convenience Store, Family Mart |
|  | **Clothing Store** | Clothing Store, Uniqlo, Uniqlo, Decathlon, Li Ning, Clover, Adidas, Adidas, Nike, Nike, Apparel Store, Zara, Gucci, Esprit, Moussy, Gap, Godiva, Mango, Only, Jewelry Store, Tiffany, Ck, Cartier, Loewe, Chow Tai Fook, Pandora, Cartier, Miumiu, Boutiques, Eyewear Stores, Shoe Stores, Hot Wind, Vans, Department Stores, Muji, Muji, Luxury Stores, Burberry, Chanel, Chanel, Dior, Dior, Prada, Armani, Hermes, Hermes, Armani, Louis Vuitton, Lv, Maxmara, Coach, Bvlgari Bosch, Discount Store, Sale, Outlet |
|  | **Cosmetic Store** | Cosmetic Store, Watsons, Beastie, Lancome, Shiseido, Kojen, Sephora, l'Occitane, Sisley, Sisley, Kate, Fresh, l'Oreal, Innisfree, Lamer, Estee Lauder, Cpb, Ysl, Terry House, Guerlain, Drug Store |
|  | **Restaurant** | Cook Shop, Restaurant, Food Shop, Dining Room, Eatery, Grandmother’s Home, Ippudo, Food Republic, Xibei, Mystic South-Yunnan Ethnic Cuisine, Lei Garden, Tang Gong, Sense 8, Secret Recipe, South Memory, Simply Thai, Heiseiya, Spicy Joint, Kwel Mun Lung, Din Tai Fung, Tea Restaurant, Lung Kee, Conrad, Blue Frog, Tsui Wah, Western Restaurant, Babela’s Kitchen, New Element, Wagas, Tasty, Papa John’s, Canteen, Hot Pot Restaurant, Dolar Shop, Faigo Hot Pot, Tan Ya Xue, Snack Bar, Snack Street, Snack Bar, KFC, KFC, Mcdonald’s, Pizza Hut, Yang’s Fried Dumplings, Fifth Avenue, Bakeshop, Bakery, Yamazaki, Dessert Shop, Häagen-Dazs, Ladym, Honeymoon, Letao, Ichidol, Paris Baguette, Mrchoi, Godiva, Food Festival |
|  | **Experience Store** | Experience Store, Apple, Evelom, Pop-Up Store, Bazaar, Marketplace |
| **Perception of Safeguarded Amenities** | **Parking** | Parking Lot, Parking Garage, Garage, Parking Spaces, Shuttle, Escalator, Elevator, Straight Stairs, Signage |
|  | **Hygiene** | Washroom, Lavatory, Wc, Toilet, Bathroom, Mirror, Trash Can |
|  | **Inquiries** | Help Desk, Service Centre, Front Desk, Robotics |
|  | **Rest** | Massage Chairs, Seats, Chairs, Stools, Rest Areas |
|  | **Health** | Mother And Baby Room, Breastfeeding Room, Gym, Wales |
| **Perception of Natural Amenities** | **Flora And Fauna** | Rabbits, Animals, Pets, Lambs, Dogs, Fish Tanks, Swans, Alpacas, Piglets, Peacocks, Farmstead, Farms, Roses, Gardens, Florists, Succulents, Greenery, Plants |
|  | **Artifacts** | Pools, Fountains, Snowflakes, Oceans, Greenery, Forests, Waterfalls, Temperature, Air, Smells |
|  | **Lights And Shadows** | Lights, Lights And Shadows, Colored Lights, Lanterns, Neon Lights, Neon Street |
| **Perception of Place** | **Transportation Routes** | Line 1, Line One, Line 9, Line Nine, Line 2, Line 4, Lin Four, Line 8, Line Eight, Line 4, Line 12, Line Twelve, Line 3, Line 10, Line Ten, Line 7, Line Seven, Line 11 |
|  | **City Roads** | Century Avenue, Republicanxin Road, Kaixuan Road, Huashan Road, North Sichuan Road, Daning Road, Loushanguan Road, Qufu Road, Dapu Road, Taikang Road, Haining Road, Huaihai Road, Fuzhou Road, Zhaojiabang Road, Xijiangwan Road, Zunyi Road, Jinko Road, South Shaanxi Road, South Huangpi Road, Nanjing Road, Nanjing Road East, Nanjing Road, Huaihai Road, West Nanjing Road, North Xizang Road, Yaohua Road, Xujiahui Road, Changning Road, Middle Xizang Road, Ruijin Road Number Two, West Lujiazui Road, Hongqiao Road |
|  | **Landmarks** | Shanghai Exhibition Centre, Shanghai Bund, Oriental Pearl Tower, Tv Tower, China Art Palace, China Pavilion, Zhongshan Park, People's Square, Ren Guang (Abbreviation For People's Square), The Bund, Xujiahui, Xintiandi, Mercedes-Benz, Riverside, Lingshi Park, Ruijin Hospital, Tianzifang, Jing'An Temple, Hongkong Plaza, Luxun Park, Huangpu River, Jinmao Mansion, Anyi Night Alley, Soccer Stadium, Sports Stadium, World Expo, Bookstore, Lujiazui, Financial District, Hongqiao Railway Station, Hongqiao Airport, Paris Spring, Parkson, Ruiou, Wu Jiao Chang, Yaohan, Shangjia, Buynow Pc Mall, Jinqiao International, Aquarium, International Conference Centre, Suzhou Creek, Metro Town |

**Notes:**

1. Consumers’ perception of specific amenities is limited to the brand or campaign name; therefore, this category of keywords has been retained.

2. The repetition of individual brand names indicates that the English expression of the brand is more commonly used in the Chinese context. Hence, this type of keyword is also retained.

3. The selection of synonyms is based on the rules of the Chinese context, which may not directly correspond to English synonyms due to differences in contextual understanding.
